# The immunomodulating effect of palmitoylethanolamide on human myeloid dendritic cells and its possible impact on Alzheimer’s disease

**DOI:** 10.3389/fimmu.2025.1664164

**Published:** 2026-01-02

**Authors:** Iliana Piccolino, Filomena Iannuzzi, Ludovica Lionetti, Benedetta Mazzonello, Valeria Barreca, Nerisa Banaj, Valentina Arezzini, Fabrizio Piras, Paola Bossù

**Affiliations:** 1 Laboratory of Experimental Neuropsychobiology, Scientific Institute for Research, Hospitalization, and Healthcare (IRCCS) Santa Lucia Foundation, Rome, Italy; 2 National Center for Global Health, Istituto Superiore di Sanità, Rome, Italy; 3 Laboratory of Neuropsychiatry, IRCCS Santa Lucia Foundation, Rome, Italy

**Keywords:** palmitoylethanolamide (PEA), monocyte-derived dendritic cells (MDDCs), immunomodulation, DC immunobiology, Alzheimer’s disease, inflammation, neurodegenerative diseases

## Abstract

Palmitoylethanolamide (PEA) is an endogenous fatty acid amide that has emerged as a promising therapeutic candidate for neurodegenerative disorders, particularly Alzheimer’s disease (AD). Recognized for its inherent anti-inflammatory, analgesic, immunomodulatory, and neuroprotective properties, PEA possesses a good potential as a novel treatment addressing neuroinflammation associated with neurodegeneration, even though its precise mechanisms of action remain to be fully understood. Dendritic cells (DCs) are specialized migratory innate immune cells that play a crucial role in initiating and regulating immune responses and inflammation in both the body and the brain. In AD, DCs display a dysfunctional, pro-inflammatory profile, suggesting their involvement in disease pathology and progression. To explore the therapeutic potential of PEA, this study investigated its effects *in vitro* on human monocyte-derived DCs under both normal and AD-like conditions. The results show that PEA exerts significant immunomodulatory effects, promoting the maturation of DCs in both healthy and disease states. Notably, PEA treatment appears to correct the dysregulated state of DCs observed in AD conditions. This study reveals a novel mechanism by which PEA modulates immune activity through its action on DCs. By restoring normal DC function in neurodegenerative settings, PEA may help reduce inflammation, highlighting its potential as a therapeutic agent for Alzheimer’s disease.

## 1 Introduction

Palmitoylethanolamide (PEA) is a naturally occurring lipid messenger from the N-acylethanolamines (NAEs) family, which is recognized for its neuroprotective effects and excellent safety profile and tolerability, suggesting it may offer potential advantages in the treatment of neurodegenerative disorders (1). Its effectiveness has been demonstrated in a number of neurodegenerative disorders that present with an inflammatory component, including Alzheimer’s disease (AD), Parkinson’s disease, and multiple sclerosis (2–5). In particular, when used *in vivo* in a triple transgenic mouse model of AD, the ultramicronized form of PEA exerts a combination of neuroprotective and anti-inflammatory effects, which have proven to help in preserving neuronal integrity and function (6, 7). In keeping with preclinical data, PEA supplementation in patients is able to reduce cognitive impairment in different neurocognitive disorders (8, 9). In addition, in various settings, both *in vivo* and *in vitro* and mostly by binding to peroxisome proliferator-activated receptors (specifically PPARα), PEA has demonstrated anti-inflammatory effects linked to its ability to modulate immune responses and reduce the release of pro-inflammatory mediators at both peripheral and central levels, interacting with the endocannabinoid system and other pathways (10–12).

A growing body of evidence shows that inflammation, both at brain and peripheral level, is crucial in AD pathogenesis (13, 14). Microglial cells, the resident immune cells with phagocytic functions, play key roles in orchestrating brain inflammation, although their exact role in neurodegeneration -either protective or damaging- is still widely debated. On the other hand, accumulating findings confirm that peripheral myeloid cells, which may be recruited into the central nervous system early during the disease development, may also participate in AD pathogenesis (15, 16). In this scenario, it is conceivable that PEA with its anti-inflammatory properties contributes to alleviating the AD inflammatory burden by acting at various levels and on different cell compartments. In fact, in addition to reduce the activation of mast cells, leading to decreased release of prostaglandins and other pro-inflammatory mediators, PEA has been shown to influence microglial activation by inducing an increase in phagocytic activity and migration in these cells. It is also reported to modulate the function of macrophages, by promoting a pro-resolvin phenotype, inhibiting the NLRP3 inflammasome/IL-1β pathways and encouraging macrophage polarization towards the anti-inflammatory M2-type phenotype (17–20). Additional studies suggest a PEA effect on modulating inflammatory responses by directly targeting T lymphocytes and affecting their differentiation (21, 22). Furthermore, since PEA exerts anti-inflammatory effects, and considering that pro-inflammatory cytokines can influence synaptic transmission, an indirect neuroprotective mechanism of PEA may lie in its ability to modulate cytokine production, thus supporting synaptic integrity and neuronal communication (23, 24).

Overall, these results encourage the study of PEA as therapeutic support in patients with AD, in keeping low inflammatory state in central nervous system (CNS) and ensuring and sustaining the balance of central and peripheral anti-inflammatory endogenous response mediated by myeloid cell populations. However, the mechanisms through which PEA modulates innate immune cells during neuroinflammation are still unclear, thus a better comprehension of its ability to target different types of myeloid cells could provide important clues to dampen neurodegeneration.

Dendritic cells (DCs), a myeloid population of professional antigen-presenting cells (APC), are crucial orchestrators of the immune response. They are involved in maintaining homeostasis also within the CNS, where they can participate in neuroprotective responses and regulate inflammation, thus contributing to control neuroinflammation (25, 26).

Generally, DCs are present in the brain parenchyma in low numbers, but they may become abundant in the inflamed brain in several neurological disease conditions, including Alzheimer’s and Parkinson’s diseases, as well as multiple sclerosis (27, 28). In particular, DCs show dysregulated functions in AD conditions, playing a potential role in disease pathogenesis, as previously reviewed (29). Briefly, in AD mice, blood-derived DC-like cells, which are increased at perivascular and leptomeningeal site, appear dysfunctional and lead to reduced T cell activation (30). Also, monocyte-derived DCs (MDDCs) obtained from AD patients, likewise MDDCs from healthy donors generated in the presence of the amyloid beta peptide (Aβ), have impaired antigen presenting activity and enhanced pro-inflammatory functions (31, 32), while circulating myeloid DC precursors of AD patients are decreased in relation to disease progression (33). Hence, since DCs are patrolling cells, which migrate to infected, damaged or diseased sites to orchestrate a protective response to insults, the abnormal accumulation of misfolded proteins (i.e. Aβ and phosphorylated tau) in AD brain may prompt to a misleading activation of DCs and to a non-resolving inflammatory response that, in turn, could exacerbate neuronal damage.

Given the above reported observations, it is reasonable to assume that the anti-inflammatory and immunoregulatory properties of PEA could influence also DC functions, thereby modulating the maladaptive immune response in AD. To investigate this previously unexplored aspect, the present study aimed to evaluate the effects of PEA on human DCs by analyzing both the phenotypic and functional characteristics of monocyte-derived dendritic cells (MDDCs) *in vitro* following PEA treatment. To extend this investigation to AD conditions, similar analyses have been also conducted on MDDCs derived from AD patients or generated in the presence of Aβ. This approach aims to further support the concept that PEA may represent a promising strategy to counteract AD neurodegeneration by targeting and modulating DC-related immune dysfunctions and neuroinflammation.

## 2 Materials and methods

### 2.1 Media, reagents and ELISA

All cell cultures were maintained in RPMI 1640 medium, containing 2mM Glutamax, 25mM Hepes buffer, 0.1M sodium pyruvate, 0.1M nonessential amino acids and 5µg/ml gentamicin (all from Invitrogen Life Technologies, Grand Island, NY), supplemented with 10% fetal bovine serum (Hyclone, Logan, UT), thereafter referred to as complete medium. Other reagents were obtained from the indicated sources: FITC-dextran and LPS (*E.coli*, serotype 055:B5) were purchased from Sigma–Aldrich (St.Louis, MO), Aβ 1–42 synthetic peptide from PolyPeptide (Strasbourg, France) and PEA from Tocris (Bristol, UK). Briefly, PEA was prepared by dissolving 10 mg PEA in 2.19 ml DMSO to obtain a 15 mM stock solution (solubility in DMSO reported as 20 mM), which was then diluted into culture medium to produce a working solution of 1.5 mM PEA in 10% DMSO. Cells were treated with this working solution to achieve a final concentration of 30 μM PEA with 0.2% DMSO in the culture medium. Additional treatment concentrations were included for dose-response: 10 μM PEA (final 0.06% DMSO) and 100 μM PEA (final 0.6% DMSO). In selected experiments DMSO alone used at the percentage corresponding to PEA concentration was indicated as “vehicle”.

Recombinant cytokines GM-CSF and IL-4 were purchased from Miltenyi Biotec (Bergisch Gladbach, Germany). Recommended pairs of specific antibodies (coating and detecting) and standards for IL-2, IL-10, IL-12 (p70), TNF-α, IL-1β and IL-6 were purchased from Endogen (Woburn, MA) and used according to the manufacturer’s instructions. IL-18 was determined by ELISA using coating antibody (clone 125-2H), detecting antibody (clone 159-12B) and standard human recombinant IL-18, all from MBL (Nagoya, Japan). The detection limit was 15 pg/ml for all the cytokines tested by ELISA.

To enable analysis of large cytokine panels using small sample volumes, the Mesoscale Discovery (MSD Gaithersburg, Maryland, USA) multiplex platform was also used. Two U-PLEX 10-plex kits (for GM-CSF, IL-2, IL-4, IL-8, IL-10, IL-12p70, VEGF-A, Fractalkine, IL-23, IL-33 and IFN-γ, IL-1β, IL-6, IL-17A, TNFα, MCP-1, G-CSF, IL-18, IL-1RA) were purchased from MSD (K15067M-2 and K151ACM-2). All reagents were provided with the MSD kits and were used according to the manufacturer’s instructions. Each 96-well plate contained 10 carbon electrodes in the bottom of each well. Prior to sample and standards addition, wells were coated with biotinylated capture antibodies bound to unique U-plex linkers, enabling spatially resolved antibody immobilization on the U-plex plate and allowing the simultaneous measurement of up to 10 cytokines per well. Detection antibodies were conjugated with electro chemiluminescent labels (MSD GOLD™ SULFO-TAG) and bound to the analytes to complete the sandwich immunoassay. The U-plex plates were loaded into a MESO QuickPlex SQ 120 plate reader and raw data were collected as electrochemiluminescence signals detected by photodetectors and analyzed using Discovery Workbench 4.0 software (MSD). A 4-parameter logistic fit curve was generated for each analyte using the standards, and sample concentrations were calculated accordingly. Equivalent results were obtained when MSD and ELISA data were compared (not shown).

### 2.2 Generation of immature and mature MDDCs

Human monocyte-derived DCs were generated from peripheral blood mononuclear cells (PBMCs) obtained by Ficoll-Hypaque gradient centrifugation either as buffy coat preparations by the byproducts of platelet pheresis from normal healthy donors kindly provided by the Transfusion Medicine Unit, San Camillo-Forlanini Hospital (Rome, Italy), or from the whole blood of patients recruited in the Santa Lucia Foundation outpatient memory clinic (Rome, Italy), in accordance with the Institutional Review Board approval. CD14^+^ blood-derived monocytes were purified by PBMCs using CD14 magnetic antibody cell sorting (CD14-MACS) positive selection (Miltenyi Biotec, Bergisch Gladbach, Germany). They were cultured at the final density of 1.5×10^6^ cells/ml for 7 days in 24-well plates (Costar, Cambridge, MA) in fresh complete medium supplemented with 50 ng/ml GM-CSF and 10 ng/ml IL-4 (34). Such cell population has been indicated throughout the study as immature MDDCs. In some cases, in order to induce maturation, LPS (200 ng/ml) has been added to immature MDDCs for the last 48 h of culture (LPS MDDC). Where indicated, Aβ 1–42 peptide (Aβ) at the final concentration of 2 µg/ml was added to the culture together with GM-CSF and IL-4. These differently generated DCs, whose characteristics are reported in more details elsewhere (31), will be hereafter referred to as AβMDDC. 30µM PEA treatment (as optimal dose, selected from concentrations ranging from 0.1 to 100µM, none of which affecting cell viability) was performed in the absence of serum for the last 48 h of culture, as well. On day 7 both immature and mature PEA-treated and -untreated cells were collected and analyzed. Supernatants were stored at −80°C until use. Cell count and viability were determined by trypan blue (Gibco) exclusion method.

### 2.3 Flow cytometry and mAbs

The following mAbs (all from BD, San Diego, CA) were used for flow cytometry analysis: anti-HLA-DR (clone L243), anti-HLA-ABC (clone G46-2.6), anti-CD80 (clone L307.4), anti-CD86 (clone 2331/FUN-1), anti-CD83 (clone HB15e), anti-CD14 (clone M5E2), anti-CD1a (clone HI149), anti-CD11c (clone B-ly6), anti-CD40 (clone 5C3). Negative controls were isotype matched mAbs (all from BD). To determine surface cell phenotype cells were washed in assay buffer (PBS, 0.5% BSA and 0.1% sodium azide), incubated with mAbs for 15 min at +4°C, washed and then analyzed by flow cytometry. All immunophenotypic analyses were performed with a FACSCalibur flow cytometer (BD Immunocytometry Systems, San Jose, CA). Data were analyzed using CellQuest software (BD; Version 3.2.1).

### 2.4 Endocytosis

Endocytic activity was assessed by incubating cells for 1 h with 1 mg/ml dextran-FITC at either +37°C or +4°C. Cells were washed three times with PBS, and dextran-FITC uptake was quantified as percentage of fluorescent, positive cells and as Mean Fluorescence Intensity (MFI). Ten thousand cells of each sample were analyzed by flow cytometry.

### 2.5 Mixed leukocyte reaction

To test the ability of DC populations to stimulate allogeneic T cell responses, a set of assays addressed to analyze mixed leukocyte reaction (MLR) were performed. PBMCs were isolated from peripheral blood of healthy donors by Ficoll-Hypaque gradient centrifugation, then naïve CD45RA+ T cells were negatively selected by naïve CD4^+^ T cell isolation kits (magnetic cell sorter, Miltenyi Biotec). The MDDC populations, both immature or mature, either generated in presence or absence of Aβ and treated or not with PEA, as appropriate, were extensively washed with PBS, then suspended in fresh complete medium and co-cultured at titrated numbers with 1×10^5^ allogeneic T cells in 96-well, U-bottom culture plates for 5 days. T cell proliferation was analyzed by flow cytometry using the fluorescent cell staining dye Carboxyfluorescein succinimidyl ester (CFSE), following manufacturer’s protocol (CellTrace™ CFSE Cell Proliferation Kit, Thermo Fisher, Waltham, MA, USA). Briefly, T cells were suspended in PBS and labeled with 10 μM CFSE. After thoroughly washing, an aliquot of stained T cells was immediately acquired by flow cytometry to set the initial fluorescence (time zero). At the end of the 5-day co-culture assay, cells were stained with anti-CD3 APC (clone UCHT1) to discriminate between T cells and MDDCs and with LIVE/DEAD Fixable Aqua Stain (Invitrogen) to determine the viability of cells prior to the fixation with PFA 1%. Eventually, the dilution of the CFSE signal was recorded by flow cytometry and reported as percentage of CFSE loss of fluorescence, which indicates the percent of stained proliferating cells. This set of analyses was performed with Cytoflex flow cytometer and data were analyzed using CytExpert software version 2.3 (Beckman Coulter, Brea, CA, USA).

### 2.6 Activation of NF-κB by western blot

Mature and immature MDDCs treated with PEA or vehicle were lysed in RIPA buffer (Merck, Darmstadt, Germany) supplemented with Halt protease inhibitor cocktail (Thermo Scientific, Waltham, MA, USA) and Halt phosphatase inhibitor cocktail (Thermo Scientific). Total protein concentration was measured by Pierce BCA protein assay kit (Thermo Scientific), and 20 µg of protein per sample were separated on a 10% acrylamide gel and transferred to a nitrocellulose membrane. Membranes were blocked for 1 hour in blocking solution (5% nonfat dry milk in Tris-buffered saline with 0.01% Tween-20) and then incubated overnight at 4°C with gentle agitation with antibodies, either anti–NF-κB p65 or anti–phospho–NF-κB p65 (Ser536) (Cell Signaling, Danvers, MA, USA). After washing, membranes were incubated with HRP-conjugated secondary antibody for 1 hour at room temperature, developed with ECL substrate (Bio-Rad, Hercules, CA, USA), and imaged using the iBright system (Thermo Fisher, Waltham, MA, USA). GAPDH was used as the loading control. Band intensities were quantified with ImageJ.

### 2.7 Subjects

The part of the study involving patients has been approved by the Institutional Ethics Committee of the Santa Lucia Foundation (Rome, Italy; approval number: OVSZK 3572-2/2015/5200). The study was performed in accordance with the local legislation and institutional requirements and in compliance with the ethical standards laid down in the 1964 Declaration of Helsinki. Ten patients with a diagnosis of probable AD were included. Inclusion criteria were: (i) diagnostic evidence of probable AD consistent with the NINCDS-ADRDA criteria (35); (ii) mild to moderate severity of dementia, defined as Mini-Mental State Examination (MMSE) score ranging from 24 to 10 (36); (iii) vision and hearing sufficient for compliance with testing procedures; (iv) laboratory values within normal limits or considered not clinically relevant by the investigator. These subjects were drug free and underwent the first clinical examination for the diagnosis of AD. All subjects were assessed with the Mental Deterioration Battery (MDB) to investigate performances in cognitive domains.

Exclusion criteria for patients included recent or current acute infection, major concurrent psychiatric illness, other severe physical illness, or a history of other significant neurological illness and/or autoimmune conditions.

All patients gave their informed consent prior to their inclusion in the study.

As above stated, part of the human samples used in this study (e.g. buffy coats obtained from healthy donors) were acquired from residual material as by-product of routine care (San Camillo Transfusion Medicine Unit, Rome) for which written informed consent for participation was not required, in accordance with the national legislation and institutional requirements.

### 2.8 Statistical analysis

Data are expressed as median and 95% CI or mean values ± SE. All statistical analyses were performed using Prism 8 software (GraphPad Software, Inc., San Diego, CA). Comparisons between groups were conducted using parametric or non-parametric tests as appropriate, on the basis of data distribution. Specifically, for non-parametric data two-tailed Mann Whitney (unpaired) or Wilcoxon signed rank (paired) tests were used. For parametric data, two-way ANOVA to examine the effects of treatment in response to different numbers of DCs on T cell proliferation or one-way repeated measures ANOVA followed by Bonferroni’s *post-hoc* test to compare multiple related conditions were used. In addition, paired or unpaired t-test were used to compare untreated and PEA-treated conditions, as appropriate. Results were considered to be statistically significant when *p*-values were ≤0.05.

## 3 Results

### 3.1 PEA effects on buffy coat-derived MDDC

In order to evaluate the outcome of PEA exposure in human DCs, we firstly obtained MDDCs from healthy donor buffy coats either in their immature and mature form, i.e. in the absence or presence of LPS treatment, respectively, according to what we have reported in a previous paper (31). Briefly, to obtain MDDCs, CD14^+^ monocytes isolated from buffy coats were cultured as described for 7 days and treated for the last 48 h of culture with 30µM PEA. The PEA treatment optimal dose of 30µM, which did not influence the viability of MDDCs, was selected on the basis of previous dose-response experiments performed with PEA concentration (**Supplementary Figure S1**).

Untreated and PEA-treated MDDCs were successively evaluated in terms of: 1) immunophenotype, assessed as specific surface marker expression levels; 2) antigen internalization ability, measured in terms of FITC-dextran endocytosis; 3) cytokine release in cell supernatant, evaluated by ELISA or MSD assay, and 4) antigen presenting competence, estimated as allogenic T cell activation capability in the MLR assay.

#### 3.1.1 Immunophenotype

**Figure 1:**
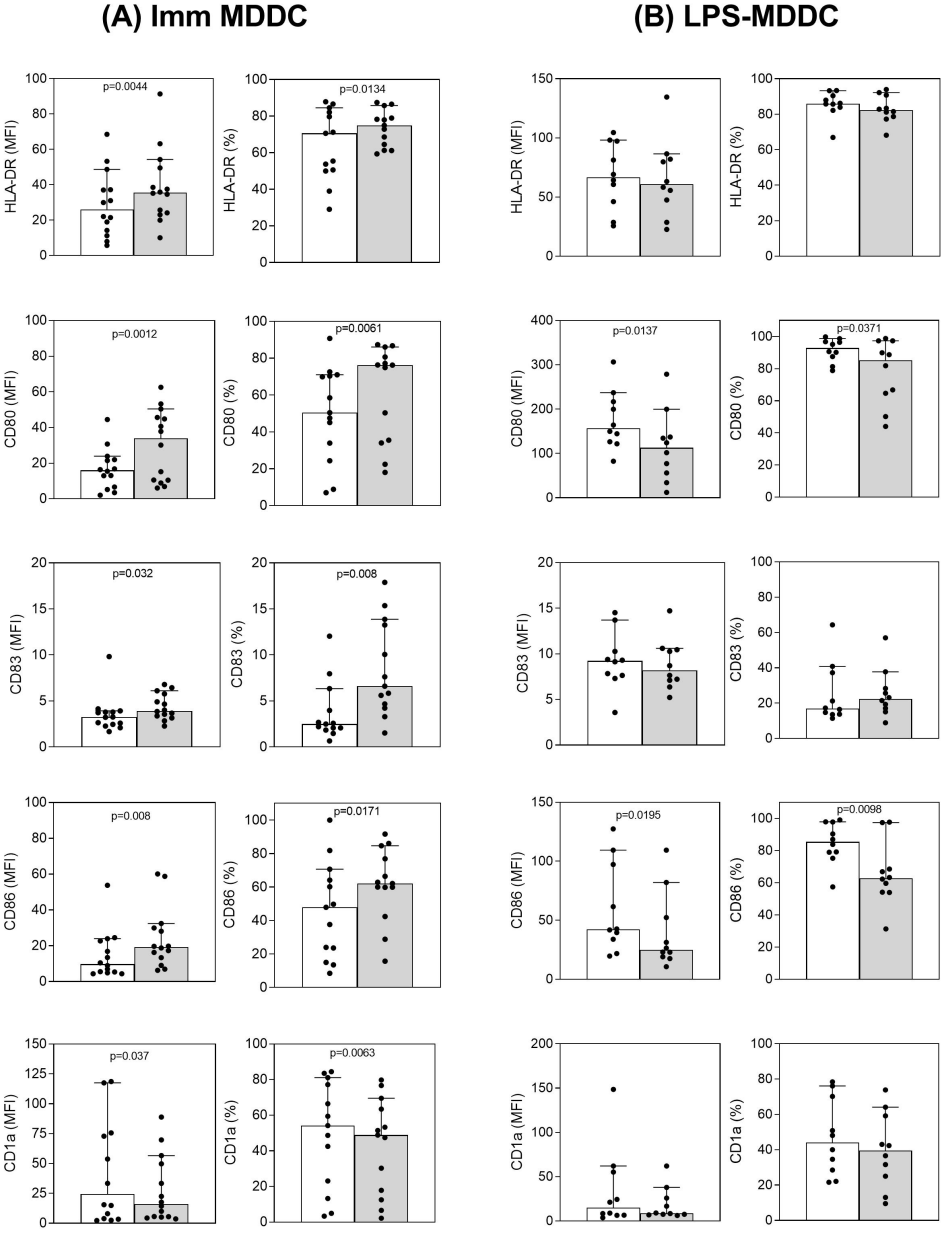
PEA-induced modulation of surface marker expression in MDDCs. Flow cytometric analysis was performed to evaluate HLA-DR, CD80, CD83, CD86 and CD1a molecule expression on immature **(A)** or mature **(B)** MDDCs, which have been untreated (empty bar) or treated with 30µM PEA (grey filled bar). For each molecule, bar graphs show the median values and 95% CI of the mean fluorescence intensity, indicated in correspondence of the fluorescent peak in each panel (MFI, left column panels), and of the percentage of positive cells (%, right column panels). Scatter plots values are included in the graphs to illustrate inter-donor variability. Data from buffy coats obtained from 14 healthy subjects are reported. Significant difference between PEA-treated vs. untreated cells is indicated.

As reported in **Figure 1**, PEA treatment is able to modulate the immunophenotype of immature MDDCs (**Figure 1A**), by inducing a statistically significant increase in the expression of HLA-DR, CD80, CD83, CD86 molecules, in terms of both measure of surface molecule abundance (MFI) and percentage of cells resulting positive (%), i.e. fraction of cells expressing the surface molecules. On the contrary, in the same conditions, CD1a molecule expression appeared reduced.

Differently, PEA did not increase the expression of the reported molecules on mature MDDCs; instead a significant decrease in CD80 and CD86 expression was observed after PEA treatment, paralleled by a trend of CD1a drop (**Figure 1B**).

Overall, this set of results indicates that PEA is able to modulate the phenotype of MDDCs, inducing a consistent upregulation of co-stimulatory molecules CD83, CD80 and CD86, as well as HLA class II molecules, mainly triggering a shift of immature MDDCs towards maturation.

#### 3.1.2 Antigen internalization ability

**Figure 2:**
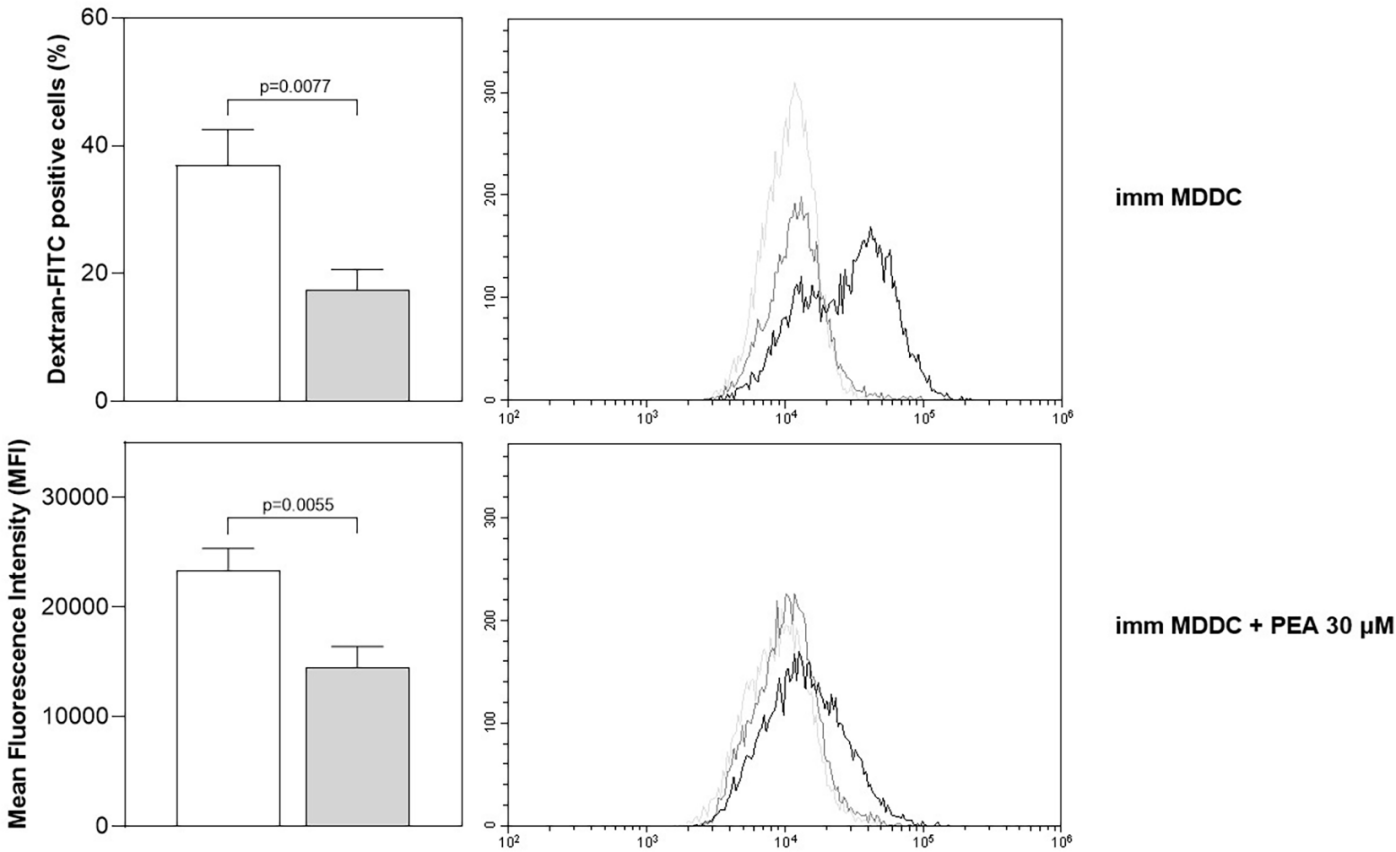
PEA-induced modulation of antigen uptake in MDDCs. Flow cytometry analysis was performed to evaluate endocytic activity of immature MDDCs. The uptake of FITC–Dextran was quantified as the percentage of FITC–Dextran–positive cells (%, upper left graph) and as delta MFI (ΔMFI, lower left graph), both calculated by subtracting the % or MFI value of the non-specific uptake at 4°C from that obtained at 37°C. Untreated cells are represented as empty bar and cells treated with 30µM PEA as grey filled bar. Data are obtained from buffy coats of 3 healthy subjects (run in triplicates) and are reported as mean ± SEM. Significant differences between PEA-treated vs. untreated cells are indicated. Representative histogram overlays illustrate the *in vitro* uptake of FITC–Dextran by immature DCs from a single donor in both untreated (upper right histogram) or 30 μM PEA-treated (lower right histogram) conditions. The FITC–Dextran endocytosis at 37°C (bold-line) is compared with the non-specific fluorescence detected at 4°C (dark-grey) and with the autofluorescence from unlabeled samples (light-grey).

One of the most important characteristics of DCs, particularly for the cells in their immature form, is the effectiveness to internalize soluble antigens. Therefore, we evaluated the impact of PEA on the capability of immature MDDCs to uptake antigens by measuring cell internalization of fluorescently-labelled dextran (dextran-FITC). The amount of dextran uptake of immature MDDCs in the absence or presence of PEA is reported in **Figure 2**, as percentage values of FITC–Dextran–positive cells and as delta MFI (ΔMFI), calculated by subtracting the % or MFI value of the non-specific uptake at 4°C from that obtained at 37°C.

In parallel with its ability to shift MDDCs toward maturity, PEA induced a reduction in antigen uptake ability in immature cells.

As expected, mature MDDCs have a poor ability to uptake antigens and the PEA treatment in these conditions has no significant effect (data not shown).

#### 3.1.3 Cytokine production

Since the production of cytokines is another distinctive ability of DCs to orchestrate the immune response, we evaluated whether PEA was able to modulate the levels of a panel of cytokines released by MDDCs in different conditions.

As summarized in **Table 1**, PEA was found to induce a statistically significant increase in TNFα, IL-10 and IL-12p70 molecules produced by immature MDDCs, as measured on cell supernatants. A trend to increase IL-1β and IL-18 was also observed in the supernatants of PEA-treated mature MDDCs, as compared to untreated cells, but these last data were not statistically significant.

**Table 1:** Cytokines produced by MDDC.

CELLS	Treatment	IL-1β	TNF-α	IL-6	IL-18	IFN-γ	IL-10	IL12p70
Immature MDDCs	Untreated	ND ^1^	3.68 ± 1.75	16.59 ± 5.71	24.37 ± 10.8	0.21 ± 0.15	123.9 ± 79.3	0.95 ± 0.53
	+ PEA	0.35 ± 0.19	**14.6 ± 4.6^2^**	28.32 ± 6.71	4.86 ± 2.33	0.06 ± 0.04	**1072 ± 564^2^**	**17.57 ± 6.85^2^**
Mature MDDCs	Untreated	113 ± 46.3	8914 ± 1515	5320 ± 242	16.92 ± 7.16	1570 ± 1048	2636 ± 1328	7692 ± 3140
	+ PEA	851 ± 228	9302 ± 1402	5646 ± 175	66.83 ± 14.5	2808 ± 1267	3989 ± 1881	10417 ± 2960

^1^Not Detectable, below the lower limit of detection of the assay;

^2^Statistically significant *versus* untreated conditions (*p* < 0.05; in bold values).

The cytokine levels are expressed as mean pg/ml (± SEM) of molecule present in cell samples obtained from 9–11 buffy coats of different healthy subjects.

#### 3.1.4 T cell activation

**Figure 3:**
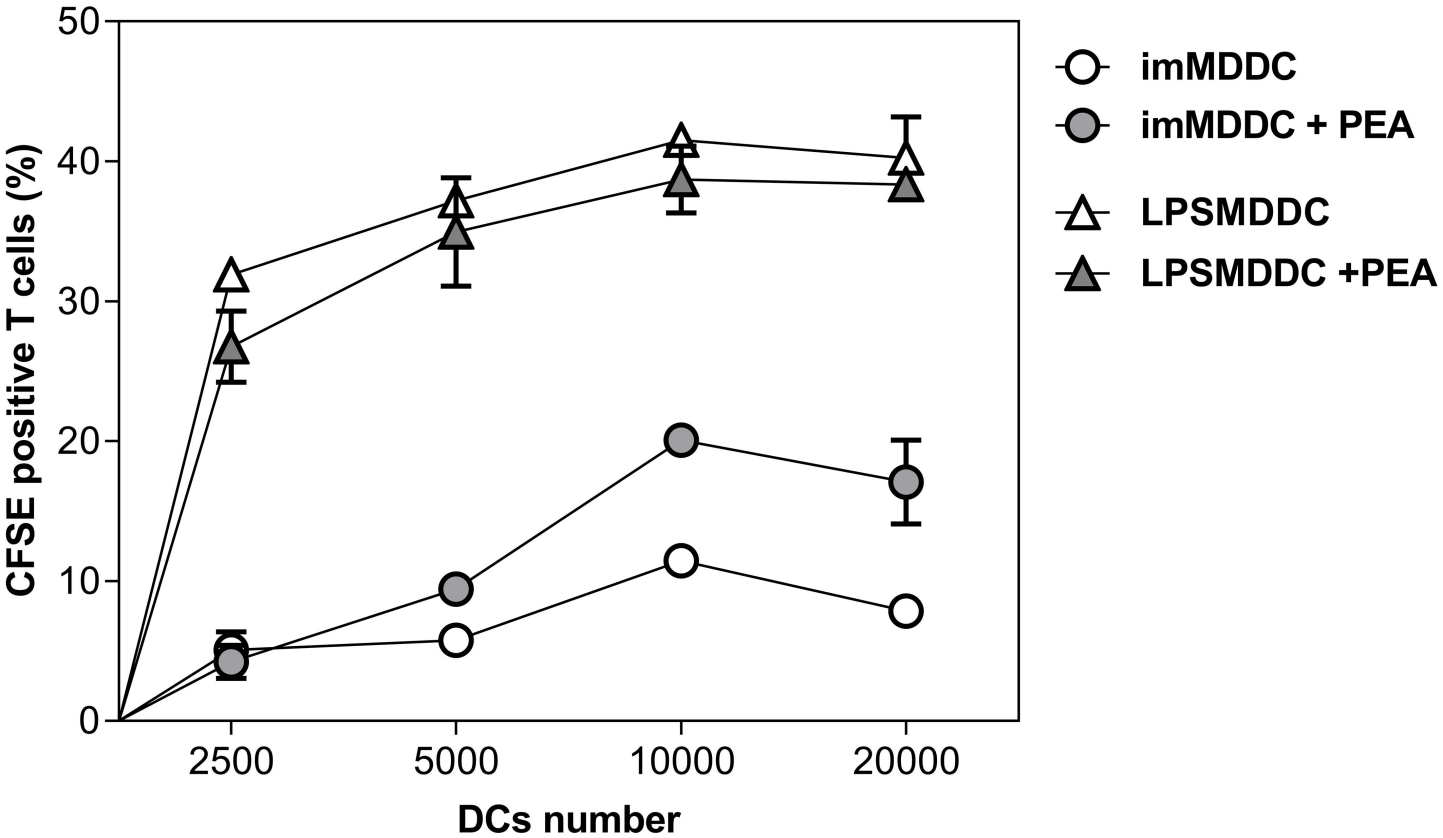
PEA-induced increased ability of immature MDDCs to stimulate allogeneic T cells. MDDCs derived from healthy donors, immature or matured with LPS, were cultured at different cell numbers with 1×10^5^ allogeneic CD4^+^/CD45RA^+^ T cells. Proliferation was determined by staining T cells with CFSE and measuring the fluorescence after the co-culture. Data are shown as mean ± SD of CFSE positive cells from one experiment run in duplicates, representative of 3 performed. A significant difference was observed between the proliferation curve obtained with untreated immature MDDCs vs. that of immature MDDCs treated with PEA 30µM. (p=0.0005).

As the main effector function of DCs is the activation of naïve T cells, we performed a mixed leukocyte reaction to test the ability of PEA to modify the MDDC-induced proliferation of alloreactive T cells following co-culture. **Figure 3** shows that immature MDDCs treated with PEA have a significantly increased ability to activate naïve T cells, as compared to untreated cells. At variance, the exposure to PEA has a trend to inhibit mature MDDC-dependent T cell proliferation, even though no significant effects of PEA have been achieved in mature DCs, which as expected, have a higher ability to activate T cells, as compared to immature cells. These results confirm the effectiveness of PEA in inducing DC maturation and promoting antigen presentation ability in the absence of other maturation stimuli, like LPS.

### 3.2 PEA effects on MDDC function under AD conditions

Since DCs appear dysregulated in AD (29), next experiments were addressed to evaluate the effects of PEA treatment on either DCs directly derived from AD patients or cultured with Aβ and recapitulating AD conditions. In fact, we have previously demonstrated that MDDCs generated *in vitro* in the presence of fibrillar aggregates of Aβ1-42 (AβMDDC) (31), as well as MDDCs obtained from AD patients (ADMDDC) (32) have both increased immature pro-inflammatory features accompanied by impaired APC ability and reduced capacity of activate naïve T cells.

#### 3.2.1 Immunophenotype

**Figure 4:**
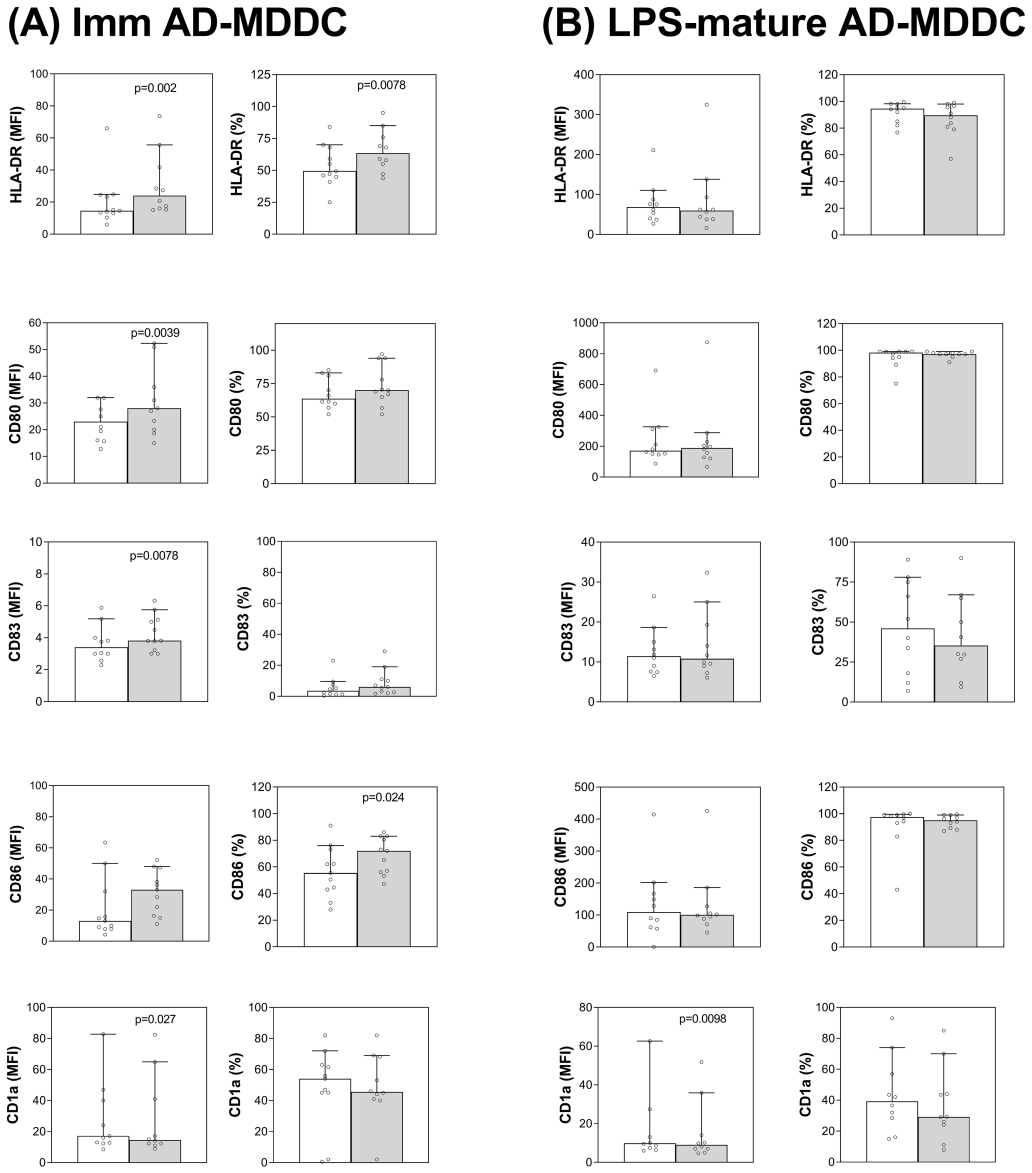
PEA-induced modulation of surface marker expression in MDDCs of AD patients. Flow cytometric analysis was performed to evaluate HLA-DR, CD80, CD83, CD86 and CD1a molecule expression on immature **(A)** or mature **(B)** ADMDDCs, which have been untreated (empty bar) or treated with 30µM PEA (grey filled bar). For each molecule, bar graphs show the median values and 95% CI of mean fluorescence intensity (MFI, left column panels), and of the percentage of positive cells (%, right column panels). Scatter plots values were included in the graphs to illustrate inter-donor variability. Data from buffy coats obtained from 8–10 AD patients (depending on specific conditions) are reported. Significant difference between PEA-treated vs. untreated cells is indicated where appropriate.

In order to confirm the DC immunomodulating properties of PEA in AD conditions, we evaluated the effect of its treatment on the surface molecule expression in MDDCs obtained from AD patients. As shown in **Figure 4**, and similarly to what reported in **Figure 1**, PEA treatment is able to modulate the immunophenotype of immature ADMDDCs (**Figure 4A**) by inducing a statistically significant increase of HLA-DR, CD80, CD83 and CD86 molecules, in terms of surface molecule abundance (MFI) or percentage of positive cells (%), or both. In the same conditions, CD1a molecule expression appeared reduced. Equally to buffy coat-derived MDDCs, mature ADMDDC phenotype is not significantly affected by PEA and the expression of the reported molecules was unchanged, a part from a reduction of CD1a (**Figure 4B**). Thus, these results confirm that PEA is able to modulate the phenotype of MDDCs also in AD conditions, inducing the upregulation of co-stimulatory molecules CD83, CD80 and CD86, as well as HLA class II molecules specifically in immature ADMDDCs, mainly triggering their shift towards maturation. In parallel, similarly to what above reported in healthy donors, PEA treatment reduces the antigen uptake ability and shows a trend to upregulate the production of some cytokines in immature MDDCs also in AD conditions (**Supplementary Figure S2**), even though these effects appear partial and not statistically significant, possibly due to the high inter-individual variability among patients.

#### 3.2.2 T cell activation by ADMDDC

As a final step to evaluate the effectiveness of PEA treatment on DC effector functions in AD patients, we measured the ability of AD-derived MDDCs to stimulate allogeneic T cell responses by means of MLR assay.

**Figure 5:**
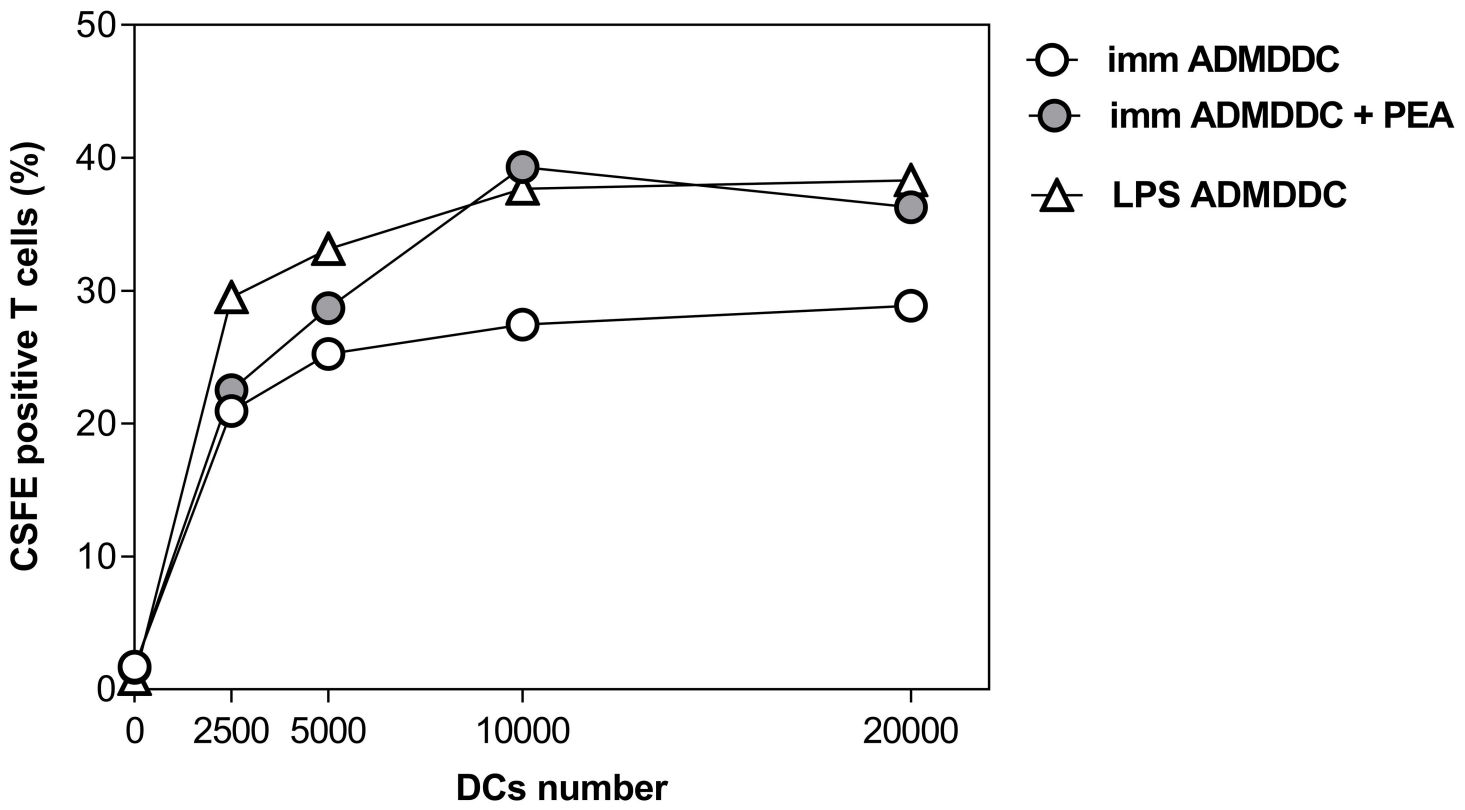
PEA-induced increased ability in immature MDDCs from AD patients to stimulate allogeneic T cells. MDDC derived from AD patients, either immature or matured with LPS, were cultured at different cell numbers with 1×10^5^ allogeneic CD4+/CD45RA+ T cells. Proliferation was determined by staining T cells with CFSE and measuring them after the co-culture. Data are shown as percentage values of CFSE positive cells from one patient, representative of 3 experiments performed with 3 different AD patients. A significant difference (p=0.018) was observed between the proliferation curve obtained with untreated DC vs. that of cells treated with PEA 30µM.

As reported in **Figure 5**, immature DCs obtained *in vitro* from AD monocytes appear to acquire increased ability to activate T cells following PEA treatment, even behaving like mature ADMDDCs and thus confirming the ability of the lipid mediator to promote DC functions and suggesting its value as immunomodulating therapeutic candidate in AD conditions.

#### 3.2.3 T cell activation by Aβ-generated MDDC

In order to assess the effects of PEA on T cell activation under AD conditions in more details, we used the before described approach to generate buffy coat-derived MDDCs in the presence of Aβ, overcoming in this way the sampling limitations related to the obtainment of MDDCs from AD patients, which otherwise would have required very high numbers of blood cells.

In particular, given that in AD conditions DCs appear dysregulated in comparison with the healthy status and AD-derived mature DCs result less efficient in inducing T cell activation (29), in the next set of experiments we focused to assess the potential of PEA treatment in restoring the impaired functions of Aβ-generated MDDC.

**Figure 6:**
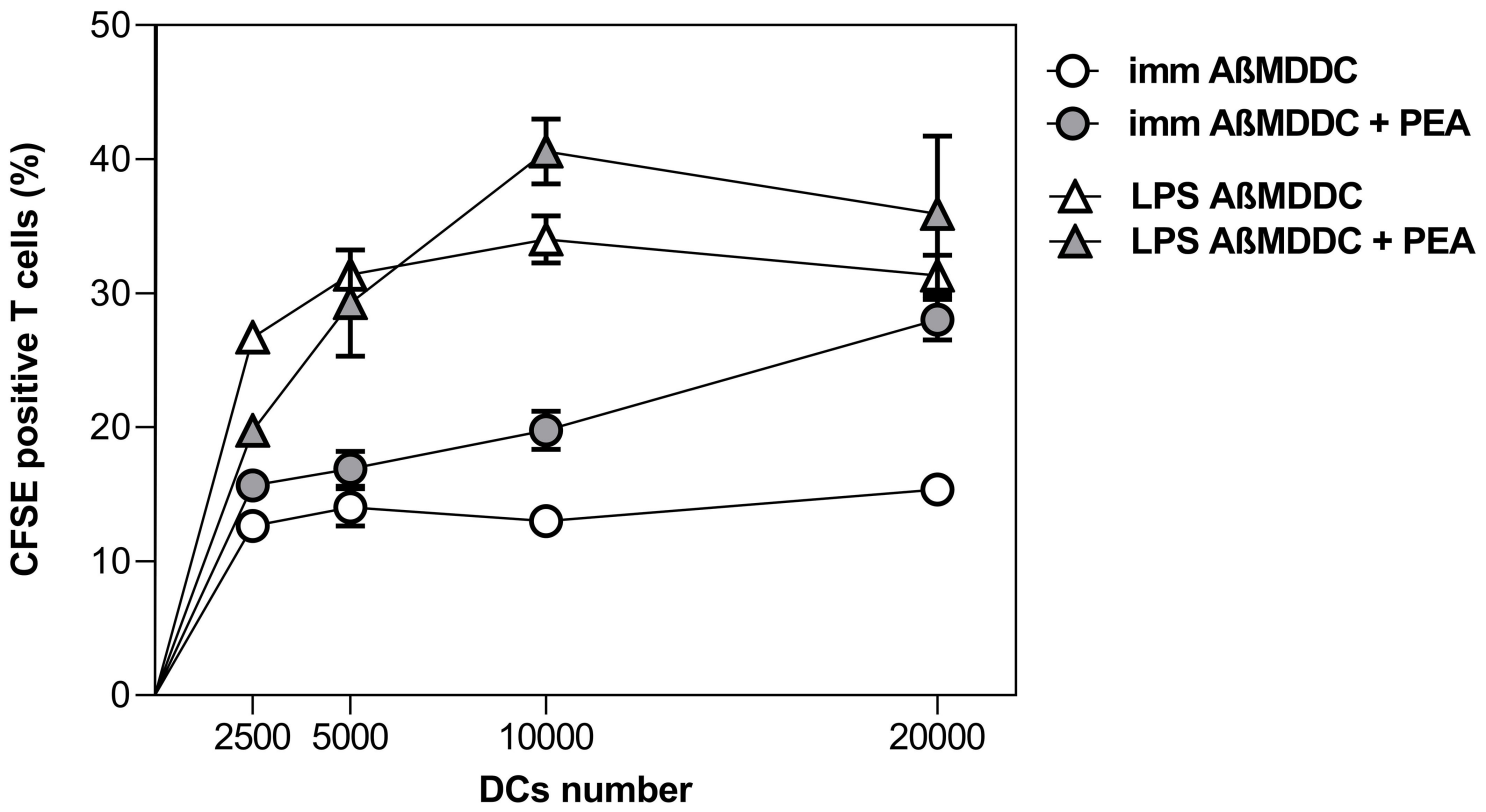
PEA-induced increased ability to stimulate allogeneic T cell proliferation in AβMDDCs. MDDCs generated in the presence of Aβ42 peptide, either immature or matured with LPS, were cultured at different cell numbers with 1×10^5^ allogeneic CD4^+^/CD45RA^+^ T cells. Proliferation was determined by staining T cells with CFSE and measuring their fluorescence after co-culture using flow cytometry analysis. Data are shown as percentage values of CFSE positive cells from one single experiment, representative of three performed with cells obtained from different donors. A significant difference was observed in immature AβMDDC between the proliferation curve obtained with untreated cells vs. that of cells treated with PEA 30µM (*p* < 0.0001).

As shown in **Figure 6**, a robust and statistically significant effect of PEA treatment on promoting T cell activation was observed regarding immature AβMDDC, confirming the ability of PEA to endorse DC maturation in AD conditions.

Of note, in this set of experiments, we also observed a PEA-dependent trend of amplification of T cell activation by mature LPS-AβMDDC, even though no statistical significance was reached in this specific case.

**Figure 7:**
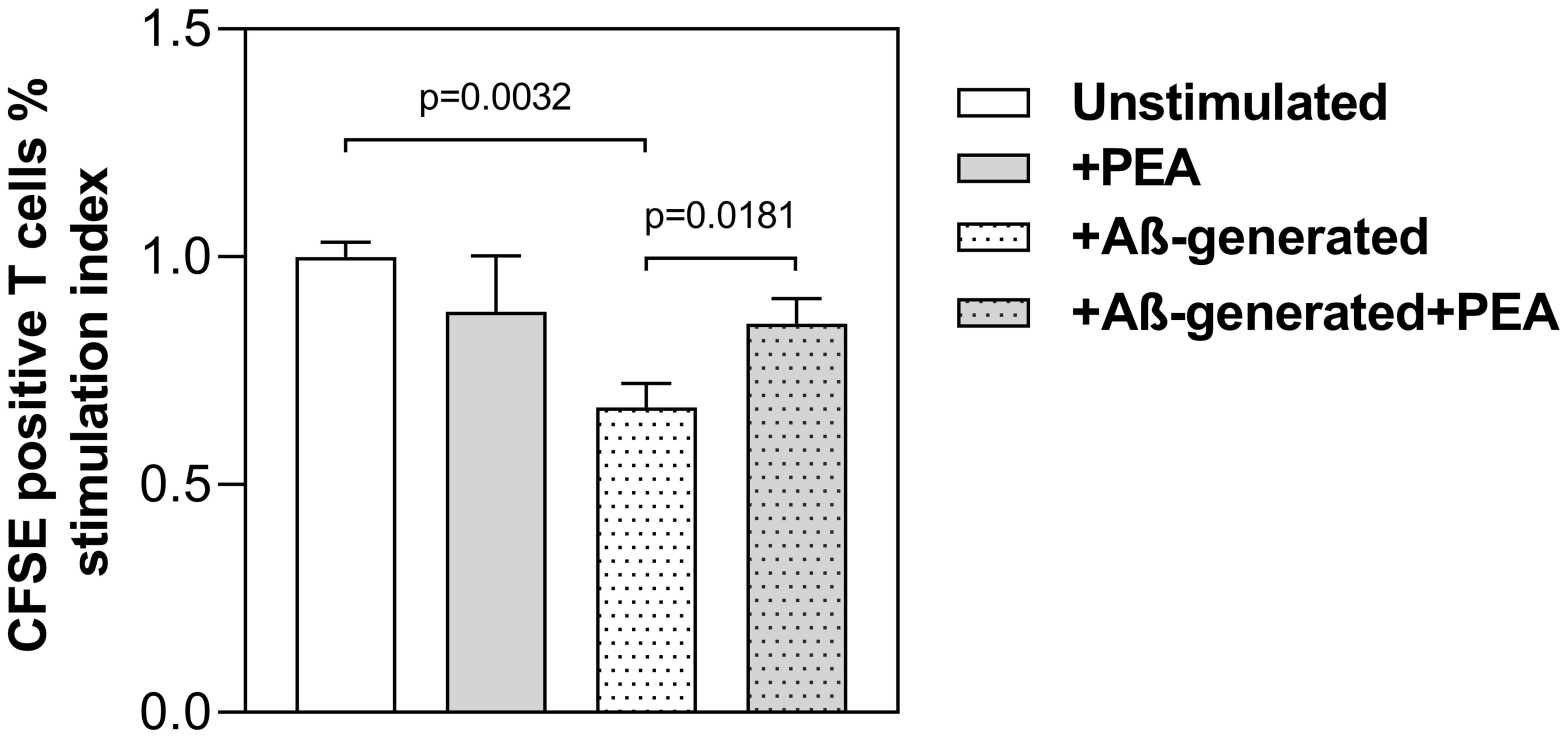
PEA-mediated ability to restore stimulation of allogeneic T cell proliferation by mature AβMDDC. 1x10^4^ MDDCs matured with LPS, either generated in the absence or presence of Aβ42 peptide and unstimulated or stimulated with PEA, were cultured with 1×10^5^ allogeneic CD4^+^/CD45RA^+^ T cells. Proliferation was determined by staining T cells with CFSE and measuring their fluorescence after co-culture using flow cytometry analysis. Data are shown as stimulation index (SI), calculated as a ratio comparing the response of stimulated to that of unstimulated DCs. Data are mean ± SEM of values from three experiments performed in duplicates with cells obtained from three different donors. The T cell proliferation induced by mature MDDCs generated with Aβ is significantly reduced as compared to normally generated mature MDDCs (*p* = 0.0032), while it is significantly increased when Aβ MDDC are treated with PEA, as compared to the untreated condition (*p* = 0.0181).

However, we were able to demonstrate this last observation by the subsequent results shown in **Figure 7**, where the magnitude of T cell response to differently treated MDDCs is reported in terms of stimulation index (SI), considering mean values from three different experiments. The graph shows that the SI reached by mature LPS-MDDCs, which as expected is reduced when DC are generated with Aβ (*p* = 0.0032), is significantly restored with PEA treatment (*p* = 0.0181).

By expressing the results as SI, we were able to reduce the effect of individual differences among participants in the MLR experiments. This allowed us to more clearly observe that PEA has beneficial effects on how DCs function, specifically in the AD context, where mature DCs have impaired antigen presentation ability. Thus, PEA appears to improve DC activity in ways that could be favorable for people with AD.

Finally, even though our study is not addressed to explore the molecular mechanisms underlying the activation effects of PEA on DCs, we obtained preliminary data suggesting that this process is mediated by the nuclear factor kappa-light-chain-enhancer of activated B cells (NF-κB) signaling pathway, as indicated by a PEA-dependent increase in NF-κB p65 phosphorylation in immature MDDCs (**Supplementary Figure S3**). Similarly, PEA treatment produced an increase in phospho-NF-κB p65 also in mature MDDCs, but without reaching the statistical significance.

## 4 Discussion

PEA is an endogenous bioactive lipid involved in and regulating many physiological processes. Its bioactivity is mimicked by exogenous PEA, which demonstrates pleiotropic effects, mostly protective, including the dampening of inflammatory response and neuroinflammation (37–39). Although the precise mechanisms by which PEA works remain unclear, its therapeutic benefits in various chronic conditions with a neuroinflammatory component, including AD, are well documented by preclinical and clinical studies (5, 7, 9, 40–42). The aim of our study was to investigate how PEA influences the differentiation and immune-regulating functions of dendritic cells under both healthy conditions and in AD, an illness in whose pathogenesis DCs may play a crucial role.

DCs serve as key immune sentinels, interpreting environmental cues to determine whether to maintain an immature or tolerogenic state or to undergo maturation in response to pathogens or danger signals, which leads to the activation of immunity. Upon maturation, DCs transform from antigen-capturing cells into potent APCs displaying on their surface antigenic peptide-loaded MHC molecules, increase the expression of costimulatory molecules such as CD80 and CD86, and secrete cytokines including interleukin-12 (IL-12) that stimulate T-cell proliferation and differentiation. These three components—antigen presentation (signal 1), costimulation (signal 2), and cytokine production (signal 3)—are all essential for effective T cell activation (43). Here, we report for the first time that an *in vitro* treatment with PEA can switch human immature DCs into maturation, ultimately driving these cells toward a more effective phenotype able to activate T cells.

In fact, we have observed that immature MDDCs, which have been treated *in vitro* for 48h with PEA are characterized by: *i*) increased expression of surface molecules related to antigen presentation; *ii*) decreased ability to uptake antigenic molecules; *iii*) modified pattern of cytokine production, and *iv*) increase of T cell activation ability. More specifically, we have shown that PEA is able to trigger immature MDDCs to significantly upregulate on their surface the expression of HLA-DR, CD80, CD83 and CD86 molecules (**Figure 1A**) and acquire an increased capacity to induce allogeneic T-cell proliferation (**Figure 3**). This finding strongly suggests that exogenous PEA plays a direct activation of antigen-presenting DCs. Furthermore, since during their maturation process, DCs down-regulate their capacity for micropinocytosis, our data showing a reduction in antigen uptake ability in immature MDDCs upon PEA treatment (**Figure 2**), confirm the promoting effect of PEA on DC maturation. It should be noted that, as expected, immature DCs have no or very low ability to present antigens and activate T cells in comparison with mature DCs, unless they are treated with PEA, as above demonstrated.

In addition, PEA-treated immature MDDCs release increased levels of TNFα, IL-10 and IL-12, as compared to untreated conditions (**Table 1**), reflecting the complex interplay of the lipid mediator with immune regulation, where each induced cytokine may modulate APC function and downstream T-cell responses. Specifically, TNFα may boost effector functions, IL-10 dampens activation, and IL-12 steers Th1 responses. Thus, the increased TNFα, IL-10, and IL-12 levels reflect a PEA-dependent dynamic regulation of immune polarization—toward inflammation, tolerance, or dysregulation—depending on contextual balances.

Overall, the concept emerging from our study is that exogenous PEA favors the transition from immature to mature DCs, independently from danger signals like LPS, thus acting as a stimulant that significantly impacts the immune system by enhancing its ability to initiate and regulate immune responses. In other words, PEA is capable of inducing dendritic cell maturation, even though it does so less effectively and with different qualitative outcomes compared to infection-derived signals.

In our setting, PEA did not increase the expression of the reported molecules on mature MDDCs. Rather, a significant decrease in CD80 and CD86 expression was observed after PEA treatment when DCs are stimulated with LPS (**Figure 2B**), in line to what previously observed in murine bone-derived dendritic cells upon stimulation of TLR signaling (44). On the other hand, this suppression of costimulatory molecules appears to be not followed by a significant modulation of T cell activation ability by mature DCs, at least under normal healthy conditions (**Figure 3**).

Still regarding the immunophenotypic profile of MDDCs modified by PEA treatment, a trend of CD1a expression drop has been also observed, and this was evident upon PEA exposure both in immature and mature MDDCs, even though no statistical significance was reached in the latter case (**Figure 1**). CD1a is a transmembrane glycoprotein expressed by DCs that plays a crucial role in presenting lipid antigens to T cells. It is possibly influenced by lipid environment; thus, it is conceivable that PEA or its metabolites could interact with CD1a or influence CD1a-mediated antigen presentation indirectly by modulating DC phenotype and function. However, no direct studies currently confirm PEA as a CD1a ligand. In addition, since CD1a^+^ MDDCs are distinguished by their increased production of inflammatory mediators, PEA treatment could possibly limit the inflammatory environment that promotes CD1a upregulation (45, 46), but further and more appropriate investigations should be performed to address this specific point. Of interest but still preliminary, our result that PEA is able to induce NF-κB p65 activation in immature MDDCs (**Supplementary Figure S3**), which is a critical step for antigen presentation and T cell activation (47), is consistent with the described effect of PEA of promoting DC maturation and enhanced T-cell priming capacity.

Most of the same changes as above described for healthy MDDCs have been also observed in MDDCs derived from AD patients. In fact, immature ADMDDC treated with PEA show a shift toward maturation both in terms of immunophenotype characterized by increased expression of the co-stimulatory molecules CD83, CD80 and CD86, as well as HLA class II molecules (**Figure 4A**), and as ability to stimulate allogeneic T cell responses (**Figure 5**).

These data are further confirmed by the use of DCs generated from Aβ-treated monocyte cultures, which represent an *in vitro* experimental paradigm able to recapitulate the modifications observed in AD-derived DCs (29). In fact, also in AβMDDC the exposure to PEA induces immature cells to promote allogenic T cell proliferation (**Figure 6**).

**Figure 8:**
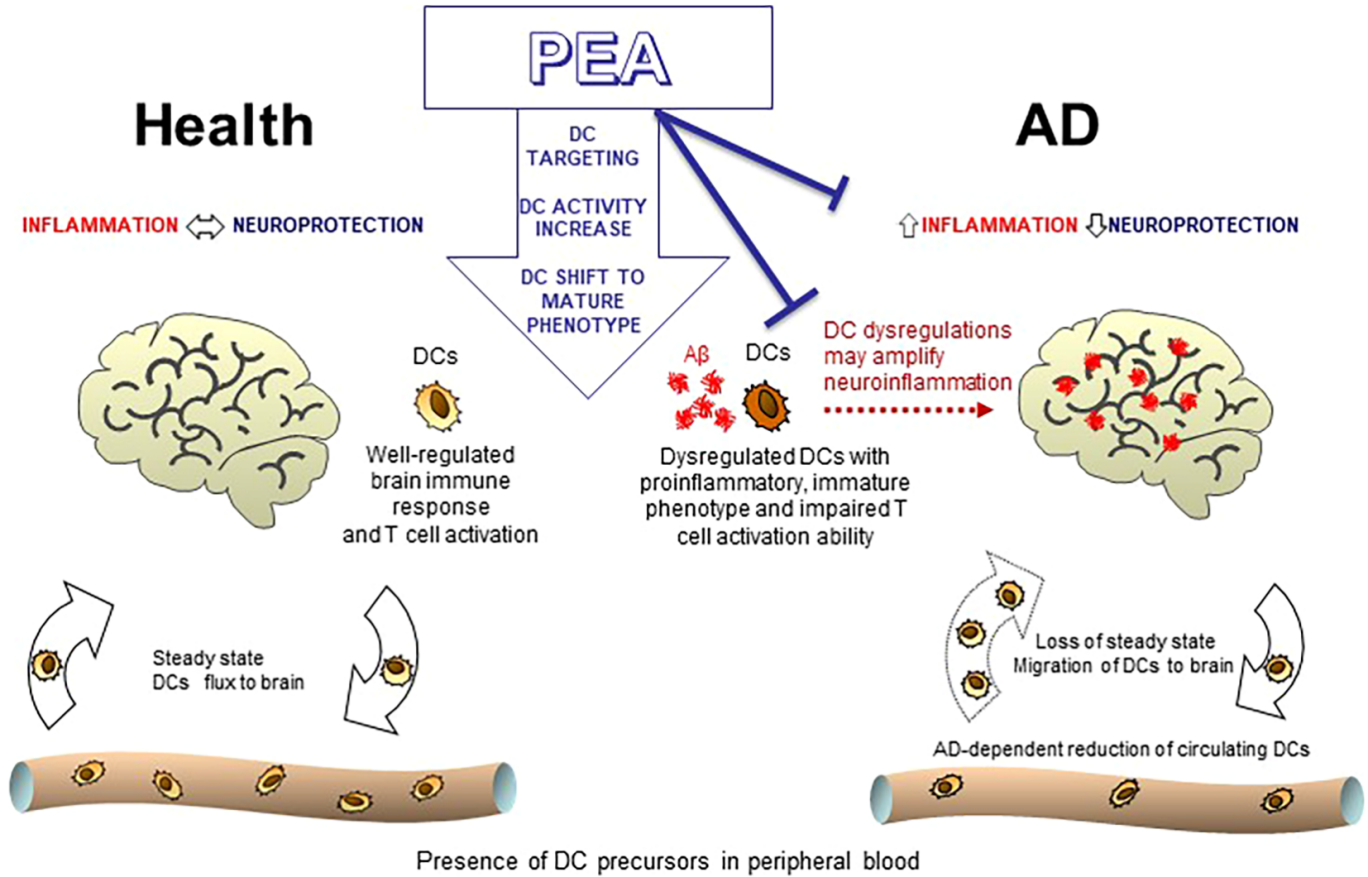
Model showing the effects of PEA on dendritic cells in healthy and Alzheimer’s disease conditions. The cartoon recapitulates our view of how PEA treatment can modulate DC activity in both healthy (left side) and AD (right side) conditions. Circulating DCs may participate in maintaining brain immune surveillance and controlling the homeostatic balance between protective and inflammatory neuroimmune processes in normal healthy conditions. During AD development, DCs could be recruited to brain, as suggested by the decline of DC *in vivo* in peripheral blood of AD patients. Upon persistent activation by aggregated Aβ, DCs may acquire a dysregulated phenotype characterized by proinflammatory, immature features and impaired T cell activation ability, as observed *in vitro*, and thus contribute to the brain inflammatory milieu. The potential therapeutic use of PEA in this context is based on its ability to shift DCs toward a more mature and effector phenotype. In fact, we observed *in vitro* that PEA promotes the maturation of DCs by upregulating co-stimulatory molecules, enhancing cytokine release, and improving antigen-presenting capacity. By targeting Aβ-activated DCs and normalizing their dysregulated phenotype, PEA may help reduce maladaptive inflammation and neuroinflammatory burden, representing a potential protective instrument against AD.

Finally, some important differences between the healthy status and the AD conditions were observed specifically regarding the APC ability of mature DCs. Although PEA appears unable to significantly modulate the expression of co-stimulatory molecules on mature ADMDDCs (**Figure 4B**), in the Aβ-generated DCs, PEA exposure increases the ability of mature DCs to activate T cells (**Figure 7**), thus restoring their decreased functionality, as it was previously observed in AD conditions (29, 31, 32). This finding confirms the promising therapeutic use of PEA in AD. Indeed, by our *in vitro* results, PEA appears to be immunoregulatory rather than purely anti-inflammatory and able to compensate for the DC functional abnormalities observed in AD and thus it is expected to be beneficial in reducing AD neuroinflammation, as summarized in the model depicted in **Figure 8**.

Overall, our results demonstrate that DCs are responsive to PEA, with exogenous PEA serving as an effective stimulant that triggers both phenotypic and functional changes in DCs, promoting their maturation from an immature, antigen-capturing state to a mature, antigen-presenting state that is crucial for initiating immune responses. Further investigation is required to understand the molecular basis of PEA’s action on DCs and to identify the specific receptors implicated in this process. However, this finding uncovers a novel mechanism of action for PEA, which is a versatile and promising lipid mediator known for its excellent safety profile. Thus, by modulating a range of immune system processes—apparently not just counteracting inflammation—PEA confirms its significant therapeutic potential particularly in AD, where a complex brain immune response contributes to neuroinflammation, neuronal damage, cognitive decline, and ultimately influences disease progression.

In summary, the translational nature of this study lies in the fact that numerous authors have highlighted the effectiveness of the nutraceutical use of PEA in counteracting certain chronic inflammatory conditions. Moreover, the absence of toxicity of this molecule and the use of formulations that enhance its efficacy and penetration, including at the cerebral level, support its use in neurodegenerative conditions. Indeed, in some AD animal models, PEA has been shown to effectively reduce neuroinflammatory parameters, particularly when used *in vivo* in its micronized formulation. However, given the poor correspondence between the inflammatory mechanisms observed in genetic animal models of AD and those characteristic of the sporadic form of the disease affecting elderly patients, the concept developed in this study—that PEA modulates DC activity, which is important in regulating chronic inflammation and neuroinflammation—, opens a new and interesting chapter for further clinical investigation.

## Data Availability

The raw data supporting the conclusions of this article will be made available by the authors, without undue reservation.
