# Supplementary material for: The immunomodulating effect of palmitoylethanolamide on human myeloid dendritic cells and its possible impact on Alzheimer’s disease
Source: Front Immunol. 2026 Jan 2;16:1664164. doi: 10.3389/fimmu.2025.1664164 (PMC12807896; doi:10.3389/fimmu.2025.1664164)
Supplement: Supplementary file 1 [file Presentation1.pdf]

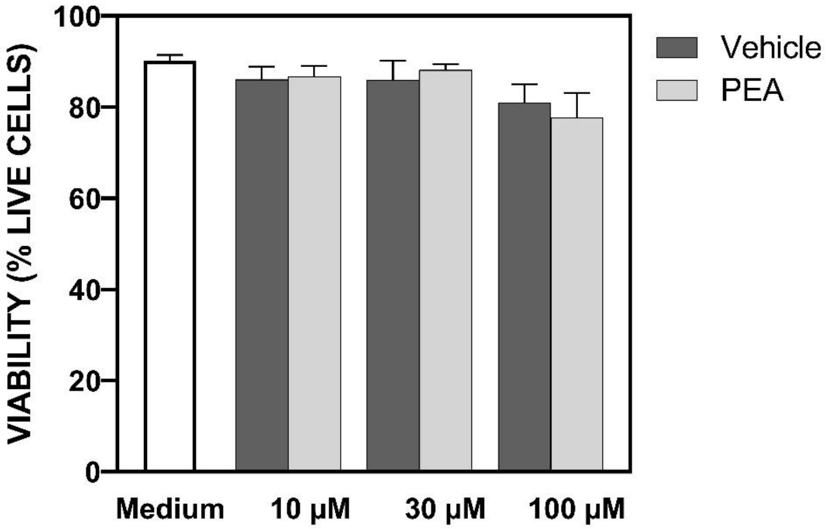

Fig. S1 *MDDC viability in PEA/vehicle treated conditions*
Immature MDDC viability was assessed by trypan blue dye exclusion and is expressed as percentage of live cells. Vehicle-treated (DMSO, dark grey bars) and PEA-treated (light grey bars) cells are shown. Data are reported as mean ± SEM from 5–9 independent experiments performed in duplicate or triplicate. No statistically significant differences are observed.

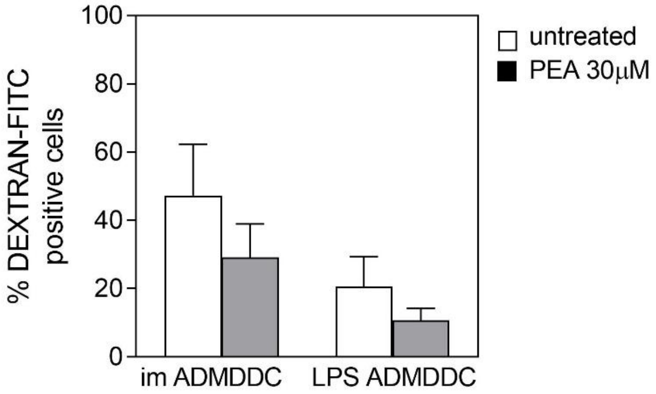

Table S1. Cytokines produced by ADMDDC

ADMDDC	IL-1β (pg/ml±SE)	TNF-α (pg/ml±SE)	IL-6 (pg/ml±SE)	IL-18 (pg/ml±SE)	MCP-1 (pg/ml±SE)	IFN-γ (pg/ml±SE)
Medium	0.5 ± 0.008	**13.41 ± 2.78**	**15.63 ± 3.49**	8.29 ± 3.83	3316 ± 2594	3.03 ± 0.23
PEA 30 μM	0.53 ± 0.14	**37.31 ± 12.76**	**40.05 ± 11.95**	9.59 ± 5.72	3768 ± 2756	2.73 ± 0.69
LPS	17 ± 3.81	29082 ± 5762	27443 ± 6773	15.34 ± 5.87	6200 ± 1942	163 ± 30.23
LPS+ PEA 30 μM	41.94 ± 14.98	30839 ± 7473	28870 ± 8740	17.74 ± 6.79	6600 ± 2407	288 ± 104.7

Fig.S2: *PEA-induced modulation of antigen uptake and cytokines production in MDDCs from AD patients*

Flow cytometry was used to assess the endocytic capacity of immature and mature MDDCs derived from AD patients. Cells were either left untreated (empty bars) or exposed to 30 µM PEA (grey filled bars). The percentage of FITC–Dextran-positive cells (%) is shown, calculated by subtracting non-specific uptake at 4 °C from uptake at 37 °C.

In the embedded Tab S1 cytokine levels in supernatant of ADMDDCs are reported as mean pg/ml (±SEM). Data are obtained from 3–10 AD subjects.

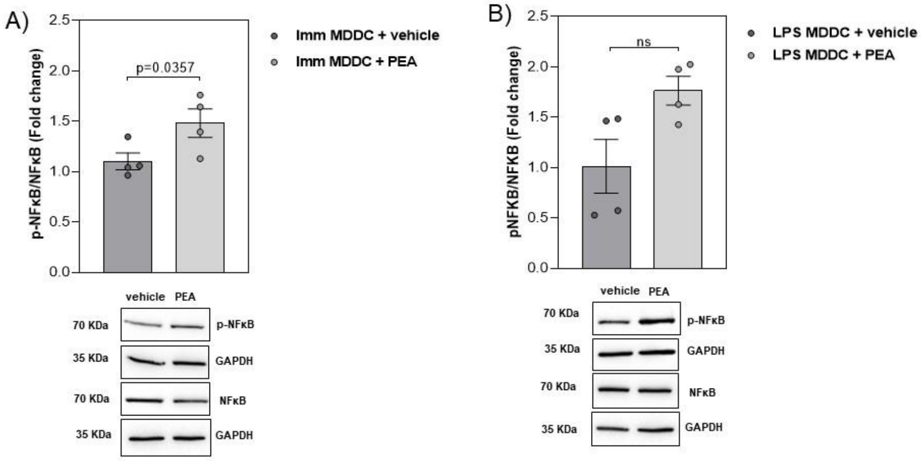

Fig.S3 *PEA treatment increases phosphorylation of NF-κB p65 in immature MDDCs*
Western blot analysis of total NF-κB p65 and phospho–NF-κB p65 (Ser536) was performed on immature (A) and mature (B) MDDCs from healthy donors treated with PEA (light gray bar) or vehicle (dark gray bar). Bars indicate fold change of PEA- or vehicle-treated samples relatively to untreated cells. Each value is the ratio between Phospho-and Total NF-κB p65 OD value, which in turn are obtained after GAPDH normalization. Data are presented as mean ± SEM from four independent experiments. Lower panels represent band intensities from a single representative experiment measured with ImageJ.
